# Pax6 interacts with SPARC and TGF-β in murine eyes

**Published:** 2012-04-18

**Authors:** Kumar Shubham, Rajnikant Mishra

**Affiliations:** Department of Zoology, Banaras Hindu University, Varanasi, India

## Abstract

**Purpose:**

To understand the mechanism of the function of paired box 6 (Pax6), a master regulator of eye development and functions, Pax6-interacting proteins were studied. It is presumed that the interaction of Pax6 with proteins in terms of morphogenesis and the maintenance of the functional anatomy of the eyes cannot be ignored. The interaction of Pax6 with matricellular protein and transforming growth factors (TGFs) is explored and presented in this report.

**Methods:**

Co-localization was studied through fluorescence microscopy. The physical interaction of Pax6 interacting proteins was explored through co-immunoprecipitation assay of samples from murine eyes.

**Results:**

It was interesting to observe the co-localization and physical interaction of Pax6, transforming growth factor-beta (TGF-β), and secreted protein acidic and rich in cysteine (SPARC) in murine eyes.

**Conclusions:**

The interaction of Pax6, TGF-β, and SPARC in murine eyes indicates that Pax6 function is regulated through TGF-β, and SPARC influences the shuttling of Pax6 via the TGF-β/Smad signaling pathway.

## Introduction

The pivotal role of paired box 6 (Pax6), a transcriptional regulator, in eyes induction was demonstrated by triggering eye formation at ectopic sites in *Drosophila* [1] and vertebrates [2]. Pax6 has been observed to localize in the nucleus, as well as in cytoplasm [3-6]. It has also been suggested that any aberration that disrupts Pax6 spatial confinement could create a diseased phenotype [7,8]. However, the cascade or hierarchy of the “eye specification gene” [9,10] is not clear.

After looking into the Pax6 interacting protein database and associated literature, the matricellular proteins and growth factors that are co-expressed with Pax6 in eyes were examined because transforming growth factor-β (TGF-β) signaling pathways have been reported to contribute maximally to maintaining the functional status of eyes [11]. Previous findings have indicated that the functional loss of TGF-β receptor type I in retinal cells results in retinal detachment [12]. This contributes to cell survival and axon guidance in the retina, as well as in the differentiation of the retinal pigment epithelium [13,14]. The Mad Homology 1 (MH1) domain of mothers against decapentaplegic homolog 3 (Smad3), a downstream target of the TGF-β signaling cascade, has been shown to interact with Pax6 [15]. Similarly, secreted protein acidic and rich in cysteine (SPARC)-producing cells have been identified in the lens, retinal pigment epithelium, and corneal epithelial cells [16-19] both in the nucleus and cytoplasm [20]. The SPARC has been implicated in cataract and corneal repair [19,21,22], and is critical for maintaining the lens transparency [23]. It has been found in vascular endothelial cells of the choroid, and blood vessels and fibroblasts of the sclera [24]. Crosstalk between TGF-β and SPARC has also been reported [25]. It is presumed that SPARC mediates the regulation of Pax6 via the TGF-β/Smad-dependent signaling pathway. In the present study, the co-localization and interaction of Pax6 with SPARC and TGF-β were explored to generate insight into molecular network Pax6 function in eyes.

## Methods

Commercial anti-Pax6 (sc-32766; Santa Cruz, Santa Cruz, CA), anti-SPARC (sc-25574; Santa Cruz), and anti-TGF-β (ab-66043; Abcam, Cambridge, UK) antibodies were used in this study. The anti-Pax6 antibody is detected at 46 kDa, the anti-SPARC antibody is recognized at 37 kDa and 25 kDa, and the anti-TGF-β antibody is detected at 13 kDa when immunoblotted. Goat anti-rabbit (HP03; Merck, Mumbai, India) and goat anti-mouse (HP021; Merck) Horse-radish peroxidase (HRP)-conjugated secondary IgG antibodies were used for Enhanced Chemiluminescence (ECL)-based detection. For immunofluorochemistry, goat anti-rabbit tetramethyl rhodamine isothiocynate (TRITC)-conjugated (RTC2; Merck), goat anti-mouse fluorescein isothiocyanate (FITC)-conjugated (FTC3; Merck) secondary IgG antibodies and 4', 6-diamidino-2-phenylindole (DAPI; Invitrogen, Eugene, OR) were used.

### Co-localization of Pax6, SPARC, and TGF-β by immunohistochemistry

Mice were anesthetized with chloroform and perfused transcardially with ice-cold PBS followed by 4% paraformaldehyde. Eyes were removed and post-fixed with 4% formaldehyde overnight, then embedded in paraffin wax. Serial sections (10 μm thick) were deparaffinized by xylene followed by rehydration through alcohol series (30%, 50%, 70%, 90%, and absolute alcohol). After antigen retrieval with 0.1% trypsin for 3–5 min, sections were blocked with 1% BSA for 30 min before double immunostaining. The first set was immunostained with anti-Pax6 (raised in mouse) + anti-TGF-β (raised in rabbit) antibodies, while the second set was immunostained using anti-Pax6+ anti-SPARC (raised in rabbit) antibodies (dilution 1:200).The sections were then incubated for 4 h at room temperature. After the incubation with primary antibodies, the sections were washed three times for 5 min with 1× PBS and probed with goat anti-mouse FITC-conjugated secondary IgG and goat anti-rabbit TRITC-conjugated secondary IgG (dilution 1:700). Experiments for negative control were performed by incubating sections without primary antibodies and counter stained with DAPI (Invitrogen,), to visualize the entire population of cells. All negative controls showed minimal or no immunofluorescence. Images were scanned using a conventional fluorescence microscope (Leica, Wetzlar, Germany). Merged images were obtained using Adobe Photoshop CS4 (Adobe Systems Incorporated, San Jose, CA) and Image J software.

### Immunoprecipitation and immunoblotting

Immunoprecipitation using mouse eye extract with anti-Pax6 and anti-TGF-β antibody was performed using the Protein-G Immunoprecipitation Kit (116622; Merck). Lysate of eyes was prepared using immunoprecipitation (IP) buffer containing 0.1% sodium dodecyl sulfate, 0.5% sodium deoxycholate, and protease inhibitor cocktail. Fifty microliters of protein-G resin slurry were suspended in three separate columns, washed twice with IP buffer, and incubated with the three different antibodies (anti-Pax6, anti-TGF-β, and anti-SPARC) at 4 °C for 60 min. The columns were again washed twice with IP buffer and 100 μl of lysate was added to the columns and incubated for another 60 min at 4 °C. The columns were washed twice with IP buffer and the protein-G resin slurry complex was re-suspended in 100 μl of sample loading buffer. The samples thus prepared were transferred in separate microcentrifuge tubes, heat denatured for 5 min and centrifuged briefly at 550× g for 20 s. The supernatant was resolved through sodium dodecyl sulfate PAGE on 10% polyacrylamide gel. The resolved proteins were then electro blotted onto polyvinylidene fluoride (PVDF) membrane. The membrane containing the pulled-down sample from anti-Pax6 was probed using the anti-TGF-β antibody, and another membrane containing the same sample was probed by the anti-SPARC antibody. Similarly, the samples immunoprecipitated with the anti-TGF-β antibody were immunoblotted using the anti-SPARC and anti-Pax6 antibodies, while the sample immunoprecipitated with the anti-SPARC antibody was probed with the anti-Pax6 and anti-TGF-β antibodies. Protein-G slurry without primary antibodies was taken as a negative control for all IP/pull-down experiments. No immunoreactivity was observed after western blot analysis. Also, liver was taken as one of control as non-Pax6 expressing tissue. The antigen-antibody complexes were detected using the ECL system.

## Results

### Co-localization and interaction of Pax6 and TGF-β

**Figure 1:**
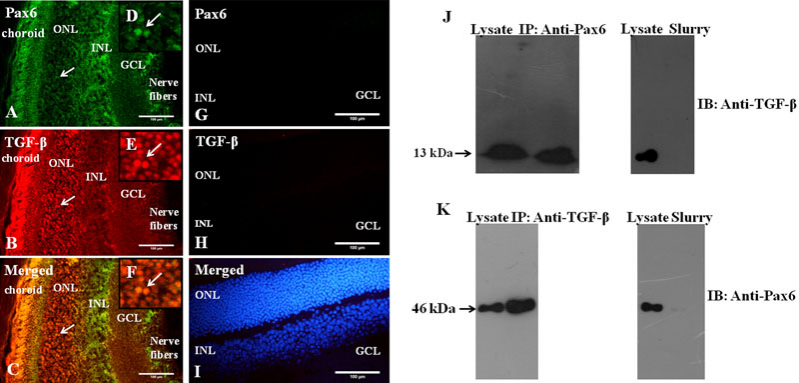
Co-localization and interaction between Pax6 and TGF-β. Fluorescence microscopy images of the retinal region of murine eyes sections showing co-localization and interaction of Pax6 and TGF-β. ONL, outer nuclear layer; INL, inner nuclear layer; GCL, ganglion cell layer. The sections were immunostained with anti-Pax6+anti–transforming growth factor–β (TGF-β). **A**: The green signal, was detected by fluorescein isothiocynate (FITC)-conjugated secondary IgG that shows the expression of Pax6, **B**: the red signals detected by tetra-methyl rhodamine isothiocynate (TRITC)-conjugated secondary IgG show the expression of TGF-β. **C**: Merged images show the co-localization of Pax6+TGF-β. Arrows in the insets (**D**, **E**, **F**) show the magnified images of the expression and co-localization. Negative control for immunostaining. **G**: without anti-Pax6 primary antibody but with FITC-conjugated secondary antibody no signals were observed, **H**: without anti-TGF-β primary antibody but with TRITC-conjugated secondary antibody no signals were observed, **I**: blue signal by staining with DAPI shows the entire population of the cells. **J**: Co-immunoprecipitation assay between Pax6 and TGF-β that is immunoprecipitated (IP) with anti-Pax6 and immunoblotted (IB) with anti-TGF- β. The next lane of the blot shows negative control of IP experiment with protein-G slurry without anti-Pax6 antibody, **K**: Immunoblotting (IB) with anti-Pax6 for immunoprecipitated (IP) with anti-TGF-β that confirms their physical interaction. The next lane of the blot shows negative control of IP experiment with protein-G slurry without anti-TGF-β antibody.

The expression of Pax6 and TGF-β was observed in the retinal layers (Figure 1A,B) of murine eyes. It was also observed that Pax6-expressing cells were positive for TGF-β expression, as highlighted in the inset (Figure 1D,E). The merged (Figure 1C,F) image indicates co-localization of Pax6 and TGF-β. Section without primary antibodies (i.e., anti-Pax6 antibody, (Figure 1G; anti-TGF-β antibody, Figure 1H), have been taken as negative control to show the specificity of the antibodies. Section was counterstained with DAPI (Figure 1I) to visualize the entire population of the cells. Moreover, physical interactions between Pax6 and TGF-β (Figure 1J), as well as TGF-β and Pax6 (Figure 1K), were confirmed by co-immunoprecipitation (Co-IP) assay. Negative controls for anti-Pax6 and anti-TGF-β antibodies by incubating protein-G slurry without primary antibody showed no immunoreactivity when immunoblotted with anti-TGF-β (Figure 1J) and anti-Pax6 (Figure 1K) antibodies, respectively.

### Co localization and interaction between Pax6 and SPARC, as well as SPARC and TGF-β

**Figure 2:**
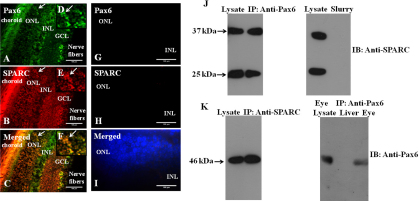
Co-localization and interaction between Pax6 and SPARC. Fluorescence microscopy images of the retinal region of murine eyes sections showing co-localization and interaction of Pax6 and SPARC. ONL, outer nuclear layer; INL, inner nuclear layer; GCL, ganglion cell layer. The sections were immunostained with anti-Pax6+anti–SPARC. **A**: The green signal was detected by fluorescein isothiocynate (FITC)-conjugated secondary IgG that shows the expression of Pax6. **B**: the red signals detected by tetra-methyl rhodamine isothiocynate (TRITC)-conjugated secondary IgG show the expression of SPARC. **C**: Merged images show the co-localization of Pax6+SPARC. Arrows in the insets (**D**, **E**, **F**) show the magnified images of the expression and co-localization. Negative control for immunostaining. **G**: without anti-Pax6 primary antibody but with FITC-conjugated secondary antibody no signals were observed, **H**: without anti-SPARC primary antibody but with TRITC-conjugated secondary antibody no signals were observed, **I**: blue signal by staining with DAPI shows the entire population of the cells. **J**: Co-immunoprecipitation assay between Pax6 and SPARC that is immunoprecipitated (IP) with anti-Pax6 and immunoblotted (IB) with anti-SPARC. The next lane of the blot shows negative control of IP experiment with protein-G slurry without antibodies. **K**: Immunoblotting (IB) with anti-Pax6 for immunoprecipitated (IP) with anti-SPARC that confirms their physical interaction. The next lane of the blot shows negative control of IP experiment with protein-G slurry with anti-Pax6 antibody, immunoblotting with anti-Pax6 using liver as non-Pax6 expressing tissue, and eye lysate as a positive control showing specificity of anti-Pax6.

**Figure 3:**
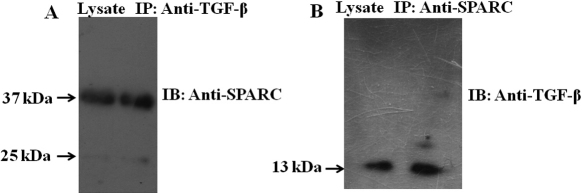
Interaction between TGF-β and SPARC. **A**: Co-immunoprecipitation assay between TGF-β and SPARC that is immunoprecipitated (IP) with anti-TGF-β and immunoblotted (IB) with anti-SPARC. **B**: Immunoblotting (IB) with anti-TGF-β for immunoprecipitated (IP) with anti-SPARC that confirms their physical interaction.

The expression of Pax6 and SPARC was observed in the retinal layers (Figure 2A,B,D,E). Double immunostaining using anti-Pax6 and anti-SPARC antibody showed immunoreactive cells. The merged (Figure 2C,F) image indicates the co-localization of Pax6 and SPARC. Negative controls without anti-Pax6 (Figure 2G) and anti-SPARC antibodies (Figure 2H) do not show immunofluorescence. The physical interaction between Pax6 and SPARC was also confirmed by Co-IP assay (Figure 2J). It was also interesting to observe the interaction between SPARC and Pax6 when cross checked (Figure 2K). Experiments for negative controls without anti-SPARC antibody showed no immunoreactivity when immunoblotted with anti-SPARC (Figure 2J). No immunoreactivity was observed in liver, a non-Pax6 expressing tissue, but eyes lysate does (Figure 2K). The physical interaction between SPARC and TGF-β (Figure 3A,B) was also confirmed by Co-IP assay.

## Discussion

The co-localization and interaction of Pax6 with SPARC and TGF-β provide direct evidence of their involvement in the cascade of Pax6 function. One previous report has indicated the cell-type-specific response of TGF-β but relatively weak DNA-binding affinity of Smad [26]. Therefore, the transcriptional efficiency of TGF-β is likely to be influenced by interactions with other transcription factors or other target proteins in a tissue-specific manner. Since the contribution of TGF-β to the development and function of murine eye is well established [11], the interaction of Pax6 with TGF-β suggests that the activity of Pax6 can be regulated via TGF-β signaling pathways. It also suggests that the biological processes regulated by TGF-β signaling pathways may involve Pax6 in a tissue-specific manner.

**Figure 4:**
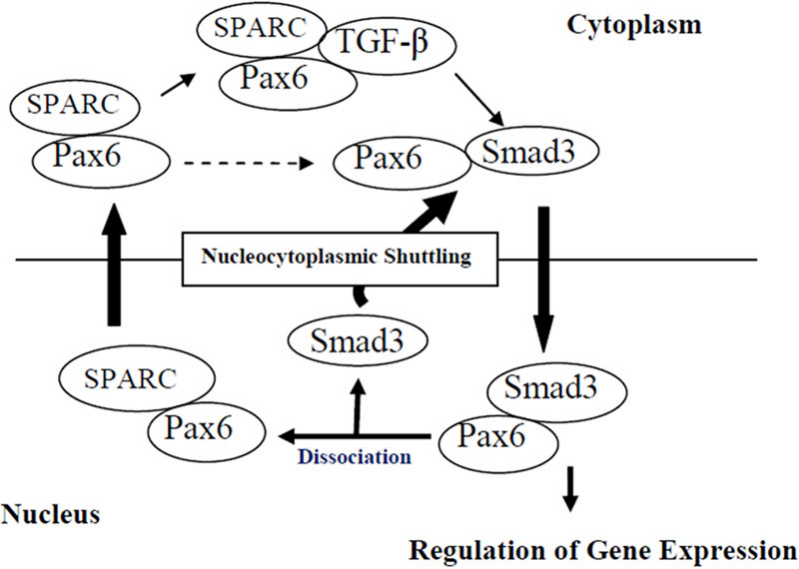
Presumptive model shows the interaction of Pax6 with SPARC that influences shuttling of Pax6 from nucleus to cytoplasm and the interaction of the Pax6/SPARC complex with TGF-β to complete the Pax6 nucleocytoplasmic shuttling cycle.

The co-localization and interaction of Pax6 with SPARC (Figure 2) indicates the involvement of a critical phenomenon of shuttling Pax6 between the nucleus and cytoplasm (Figure 4). It is likely that Pax6-associated phenotypes of the lens, retina, cornea, and iris are mediated through SPARC [16-22]. Moreover, in agreement with previous work, which found that there is a functional intersection between the pathways activated by TGF-β and SPARC [25], we conducted another set of experiments to investigate the interaction between SPARC and TGF-β (Figure 3A,B).The crosstalk between TGF-β and SPARC also supports the claim that there is SPARC-mediated regulation of Pax6 in the TGF-β signaling pathway. The interaction between TGF-β and SPARC indicates that the biological processes involving SPARC may also be regulated by TGF-β signaling pathways in murine eyes.

These findings are presumed to provide a novel regulatory mechanism of Pax6 in the murine eye through interaction-mediated translocation or compartmentalization. Smad3, a downstream target of TGF-β, also translocate rapidly in the nucleus in response to TGF-β-mediated activation [27-29]. Pax6, associated with Smad3 [15], may be translocated to the nucleus along with Smad3. Inside the nucleus, SPARC interacts with the Pax6/Smad3 complex and facilitates the regulation of Pax6-induced genes, since the binding affinity of Smad is relatively weak [26]. The nuclear retention of the Pax6/Smad3 complex may also be due to interaction with SPARC. Reports have also suggested that the Smad complex dissociates in the nucleus and is exported to the cytosol [30,31]. In the cytoplasm, the Pax6/SPARC complex may stimulate TGF-β signaling, completing the Pax6 nucleocytoplasmic shuttling cycle (Figure 4).

As a result of the considerations discussed above, our data suggest that the co-localization and interaction of Pax6 with TGF-β (Figure 1) and SPARC (Figure 2) induce the interaction-mediated regulation of Pax6 via TGF-β/Smad-dependent signaling pathways (Figure 4). It is presumed that being a transcriptional regulator, a major fraction of Pax6 remains localized in the nucleus, and a fraction of Pax6 interacts with compartment-specific nuclear or cytoplasmic proteins in Pax6-expressing tissues. The nuclear distribution of Pax6 might be due to the high-affinity interaction between Pax6 and other transcription factors in the nucleus, as well as the matricellular protein SPARC.

## References

[1] Halder G, Callaerts P, Gehring WJ (1995). New perspectives on eye evolution.. Curr Opin Genet Dev.

[2] Chow RL, Altmann CR, Lang RA, Hemmati-Brivanlou A (1999). Pax6 induces ectopic eyes in a vertebrate.. Development.

[3] Carrière C, Plaza S, Caboche J, Dozier C, Bailly M, Martin P, Saule S (1995). Nuclear-Localization Signals, Dna-Binding, and Transactivation Properties of Quail Pax-6 (Pax-Qnr) Isoforms.. Cell Growth Differ.

[4] Zhang Y, Ferreira HB, Greenstein D, Chisholm A, Emmons SW (1998). Regulated nuclear entry of the C-elegans Pax-6 transcription factor.. Mech Dev.

[5] Mishra R, Gorlov IP, Chao LY, Singh S, Saunders GF (2002). PAX6, paired domain influences sequence recognition by the homeodomain.. J Biol Chem.

[6] Tripathi R, Mishra R (2010). Interaction of Pax6 with SPARC and p53 in Brain of Mice Indicates Smad3 Dependent Auto-regulation.. J Mol Neurosci.

[7] Glaser T, Jepeal L, Edwards JG, Young SR, Favor J, Maas RL (1994). PAX6 gene dosage effect in a family with congenital cataracts, aniridia, anophthalmia and central nervous system defects.. Nat Genet.

[8] Schedl A, Ross A, Lee M, Engelkamp D, Rashbass P, VanHeyningen V, Hastie ND (1996). Influence of PAX6 gene dosage on development: Overexpression causes severe eye abnormalities.. Cell.

[9] Kumar JP (2001). Signalling pathways in Drosophila and vertebrate retinal development.. Nat Rev Genet.

[10] Gehring WJ, Ikeo K (1999). Pax 6: mastering eye morphogenesis and eye evolution.. Trends Genet.

[11] Lang RA (2004). Pathways regulating lens induction in the mouse.. Int J Dev Biol.

[12] Honjo Y, Nagineni CN, Larsson J, Nandula SR, Hooks JJ, Chan CC, Karlsson S, Kulkarni AB (2007). Neuron-specific TGF-beta signaling deficiency results in retinal detachment and cataracts in mice.. Biochem Biophys Res Commun.

[13] Liu J, Wilson S, Reh T (2003). BMP receptor 1b is required for axon guidance and cell survival in the developing retina.. Dev Biol.

[14] Trousse F, Esteve P, Bovolenta P (2001). BMP4 mediates apoptotic cell death in the developing chick eye.. J Neurosci.

[15] Grocott T, Frost V, Maillard M, Johansen T, Wheeler GN, Dawes LJ, Wormstone IM, Chantry A (2007). The MH1 domain of Smad3 interacts with Pax6 and represses autoregulation of the Pax6 P1 promoter.. Nucleic Acids Res.

[16] Mishima H, Hibino T, Hara H, Murakami J, Otori T (1998). SPARC from corneal epithelial cells modulates collagen contraction by keratocytes.. Invest Ophthalmol Vis Sci.

[17] Yan Q, Sage EH, Hendrickson AE (1998). SPARC is expressed by ganglion cells and astrocytes in bovine retina.. J Histochem Cytochem.

[18] Magee RM, Hagan S, Hiscott PS, Sheridan CM, Carron JA, McGalliard J, Grierson I (2000). Synthesis of osteonectin by human retinal pigment epithelial cells is modulated by cell density.. Invest Ophthalmol Vis Sci.

[19] Yan Q, Clark JI, Sage EH (2000). Expression and characterization of SPARC in human lens and in the aqueous and vitreous humors.. Exp Eye Res.

[20] Yan Q, Weaver M, Perdue N, Sage EH (2005). Matricellular protein SPARC is translocated to the nuclei of immortalized murine lens epithelial cells.. J Cell Physiol.

[21] Berryhill BL, Kane B, Stramer BM, Fini ME, Hassell JR (2003). Increased SPARC accumulation during corneal repair.. Exp Eye Res.

[22] Kantorow M, Huang Q, Yang XJ, Sage EH, Magabo KS, Miller KM, Horwitz J (2000). Increased expression of osteonectin/SPARC mRNA and protein in age-related human cataracts and spatial expression in the normal human lens.. Mol Vis.

[23] Kantorow M, Horwitz J, Carper D (1998). Up-regulation of osteonectin/SPARC in age-related cataractous human lens epithelia.. Mol Vis.

[24] Gilbert RE, Cox AJ, Kelly DJ, Wilkinson-Berka JL, Sage EH, Jerums G, Cooper ME (1999). Localization of secreted protein acidic and rich in cysteine (SPARC) expression in the rat eye.. Connect Tissue Res.

[25] Gotoh N, Perdue NR, Matsushima H, Sage EH, Yan Q, Clark JI (2007). An in vitro model of posterior capsular opacity: SPARC and TGF-beta2 minimize epithelial-to-mesenchymal transition in lens epithelium.. Invest Ophthalmol Vis Sci.

[26] Mu Y, Gudey SK, Landström M (2012). Non-Smad signalling Pathways.. Cell Tissue Res.

[27] Attisano L, Wrana JL (2000). Smads as transcriptional co-modulators.. Curr Opin Cell Biol.

[28] Massagué J, Wotton D (2000). Transcriptional control by the TGF-beta/Smad signaling system.. EMBO J.

[29] ten Dijke P, Miyazono K, Heldin CH (2000). Signaling inputs converge on nuclear effecters in TGF-beta signaling.. Trends Biochem Sci.

[30] Inman GJ, Nicolas FJ, Hill CS (2002). Nucleocytoplasmic shuttling of Smads 2, 3, and 4 permits sensing of TGF-beta receptor activity.. Mol Cell.

[31] Xu L, Alarcon X, Col S, Massague J (2003). Distinct domain utilization by Smad3 and Smad4 for nucleoporin interaction and nuclear import. J Biol Chem.

